# Socioeconomic hierarchy and health gradient in Europe: the role of income inequality and of social origins

**DOI:** 10.1186/s12939-015-0263-y

**Published:** 2015-11-14

**Authors:** Louis Chauvel, Anja K. Leist

**Affiliations:** University of Luxembourg, PEARL Institute for Research on Socio-Economic Inequality, Campus Belval, 11, Porte des Sciences, L-4366 Esch-sur-Alzette, Luxembourg

**Keywords:** Health inequalities, Multilevel models, Social origins, Comparative research, Health inequity

## Abstract

**Background:**

Health inequalities reflect multidimensional inequality (income, education, and other indicators of socioeconomic position) and vary across countries and welfare regimes. To which extent there is intergenerational transmission of health via parental socioeconomic status has rarely been investigated in comparative perspective. The study sought to explore if different measures of stratification produce the same health gradient and to which extent health gradients of income and of social origins vary with level of living and income inequality.

**Methods:**

A total of 299,770 observations were available from 18 countries assessed in EU-SILC 2005 and 2011 data, which contain information on social origins. Income inequality (Gini) and level of living were calculated from EU-SILC. Logit rank transformation provided normalized inequalities and distributions of income and social origins up to the extremes of the distribution and was used to investigate net comparable health gradients in detail. Multilevel random-slope models were run to post-estimate best linear unbiased predictors (BLUPs) and related standard deviations of residual intercepts (median health) and slopes (income-health gradients) per country and survey year.

**Results:**

Health gradients varied across different measures of stratification, with origins and income producing significant slopes after controls. Income inequality was associated with worse average health, but income inequality and steepness of the health gradient were only marginally associated.

**Conclusions:**

Linear health gradients suggest gains in health per rank of income and of origins even at the very extremes of the distribution. Intergenerational transmission of status gains in importance in countries with higher income inequality. Countries differ in the association of income inequality and income-related health gradient, and low income inequality may mask health problems of vulnerable individuals with low status. Not only income inequality, but other country characteristics such as familial orientation play a considerable role in explaining steepness of the health gradient.

## Background

Health is to a large extent determined by social class and socioeconomic position, henceforth referred to as health inequalities. This health stratification is a result of education, occupation and income producing differences in outcomes of morbidity and mortality, however differing depending on which stratification measure is used [1, 2]. Possible mechanisms between socioeconomic position and differences in health involve health behaviors, affordability of healthy life style and health care, absence of physically demanding work and hazardous working conditions or distress, but also reverse causation from bad health into low socioeconomic position [1–3]. Indeed, the associations of socioeconomic position and health are so strong that several researchers have put forward the hypothesis that social standing may indeed by the fundamental cause for health [4, 5]. What is even more intriguing, not only current socioeconomic position of the individual, but also socioeconomic position during childhood, i.e., social origins such as parental education and occupation affect health [6–8]. This intergenerational transmission of health via status may work through social class exposures and intergenerational reproduction of status, but studies show that intergenerational transmitted health via parental social status partly remains after controlling for adult social status [7]. Possible mechanisms in the process of transmission of health via parental socioeconomic status include stress caused by financial insecurity, material resources, and acquiring different sets of health behaviors and lifestyle from one’s parents (smoking, amount of physical activity etc. [6, 7]. What has rarely been investigated in this context is the contribution of social origins to health in a comparative perspective. It is highly likely that more meritocratic societies may produce lower origins-health gradients, or that higher income inequality may contribute to steeper income-health gradients. We are particularly interested in how contextual-level income inequality and level of development may influence the association between health and income, and health and social origins across a sample of high-income countries. In order to assess the income- and social origins-health gradient in detail and to relate these gradients to contextual variables, this contribution will introduce a methodological tool, the logit rank transformation, to assess social gradient with high sensitivity to small differences in socioeconomic resources such as income and social origins, and sensitivity to rank differences. Logit rank transformation will be used to assess impact of income and of social origins on health compared to other dimensions of social hierarchy, notably income, but also occupational class and education. The advantage of the logit rank transformation of income and social origins is that it produces a continuous comparable health gradient net of the underlying origins and income distribution, able to report the difference in health status from bottom to top social standing (steepness of the gradient) and convenient to be linked to further information, in this study contextual-level income inequality and level of economic development.

After assessing the association of health with income- and social origins across countries, net of the underlying origins and income distributions, the health gradients are investigated for associations with income inequality and levels of economic development. We assumed that higher income inequality produces steeper health gradients both of social origins and income, in line with Wilkinson and Pickett (2010) who have been arguing that equality is better of every member of the society [9]. Following this line of argumentation, a number of studies has provided evidence for positive associations of level of economic development with health, and negative associations of income inequality (less egalitarianism) with health [10–18]. In this respect, one notices a lack of comparative studies on the intergenerationally transmitted health gradients, i.e., social origins (not of *current* measures of socioeconomic class and position). Whereas evidence points to the important role of social origins for health [6–8, 19, 20], to our knowledge this is the first study to relate origins-health gradients to contextual information of level of income inequality and economic development. Wilkinson and Pickett (2010) argue that more income equality within a society would benefit everyone, even the richest. In this line of argumentation, more equality would benefit individuals from all social origins, from those experiencing childhood poverty to those with a well-off childhood socioeconomic position.

The first contribution of this study is thus to conceptualize detailed health gradients by using logit rank-transformed measures of income and social origins, net of the range of income (i.e. income inequality) within a country. This way it is possible to assess if health gradients are linear even at the top and bottom of the income and social origins distributions. The second contribution is to relate these health gradients to contextual information of income inequality and level of economic development. This will be achieved by making use of random slopes derived from multilevel models in order to estimate country-specific levels and slopes of health (net comparable health gradients), and to link them to the country-level characteristics level of economic development and income inequality. It is hypothesized that health gradients of income and social origins are stronger in more unequal countries. The research questions were studied with use of the 2005 and 2011 EU-SILC data of a total of 18 countries.

## Method

### Data

EU-SILC data of 2005 and 2011 with the module on Intergenerational transmission of poverty/disadvantages were used of 18 countries (AT, BE, CZ, DE, DK, ES, FI, FR, HU, IS, IT, LU, NL, NO, PL, PT, SE, UK) and respondents aged 25–65 years (the Intergenerational module in 2005 was only used for respondents <66, the module in 2011 was only used for respondents <60), with a sample of *N* = 299,770 individuals after data cleaning.

*Self-rated health* was used as dependent variable, recoded such that higher values reflect better health, with a range from 2 (“very good”) to −2 (“very bad”), centered and weighted.

*Age* at the end of the income reference period was centered at age 25 and squared by 10 to obtain coefficients of age in decades. The age coefficients can be interpreted as change in self-rated health per decade of age. *Gender* was centered and coded such that 1 refers to female gender.

*Stratification variables*. Stratification variables were *social origins*, i.e., parents’ socioeconomic situation indexed by mothers’ and fathers’ education (ISCED), and fathers’ occupation (EGP class). Missing values were partly replaced with the dominance approach [21], if still missing cases were assigned a separate value to limit attrition due to missing data. The three variables were factor analyzed, and the first axis of the MCA, presenting scores from lowest to highest social resources of parents, was logit-ranked to reflect a hierarchical variable of social origins. Further, stratification was assessed by *education* (ISCED), recoded in three categories, *occupation* (EGP class) in six categories, and *income position* indexed by equivalized disposable household income and logit ranked.

*Country-level variables*. Log level of living and the Gini index were computed and centered on the base of income per country in 2005 and in 2011.

### Logit rank transformation

Stratification variables income, principal factor score of social origins, and the outcome self-rated health were logit-rank transformed. Logit-rank transformation employs Champernowne’s distribution [22, 23] as extension of the tool log of Tam’s PSI [24]. In short, logit rank can be applied to any non-continuous (ordinal) variable and is useful to standardize variables in comparative inequality contexts, by exploiting within country-variation (e.g. comparing bottom 5 % of country A to bottom 5 % of country B). In the context of distributional analysis, it provides a “net of distributional change” relative reference position of individuals and of groups. Logit rank procedure is implemented in Stata as abg.ado [22]. Running the analyses with the ordinal measure of self-rated health led to similar results, however due to the large sample model estimation convergence was not optimal. Therefore we present estimations based on the logit rank transformed outcome of health.

ABG rescales a continuous variable such as income i in a logistic distribution (first i is transformed in its “fractional rank” or “continuous percentile” p in]0,1[and second in logit(p) = ln(p/(1 − p)). Thus, income distributions of different intensities of inequality are transformed in comparable standardized distributions and allow comparisons of countries where the baseline of comparison is relative percentile position, not between 0 and 1 (with problematic border effects), but between − infinite to + infinite, with the resulting logistic distribution being useful for various types of regressions including OLS or mixed multilevel models. Earlier assessments of the magnitude of health inequalities such as ratio of low vs. high, Gini-like coefficients, population-attributable risk [25] or the relative index of inequality [26] have different shortcomings. Gini-like coefficients do not reflect the hierarchical nature of socioeconomic status. The relative index of inequality does not differentiate between if there is a large effect of socioeconomic position on the health outcome or if there are large inequalities in socioeconomic variables themselves [25]. Most of those measures produce only crude measures of inequalities, and cannot be linked to further information. In contrast, a logit rank transformed measure of stratification such as income produces a gradient with information about its linearity and steepness (difference between bottom and top of the income distribution) while being net of the underlying range of income distribution.

An additional advantage of the logit rank procedure is to allow a more accurate analysis of the farther tails and within more crude measures of stratification such as EGP class. Traditionally EGP classes are interpreted as ordered groups, assuming homogeneity within classes. In reality, class and income positions both contribute in terms of cumulative advantages. In EU-SILC data for higher and lower service classes, health status is continuously improving with higher income within classes. Health status of median income higher level service class is comparable to top decile of income in lower level service class, when graphing health and logit rank from 0 (median) to 5 (near quantile 99.5 %, see Appendix Fig. 7).

### Multilevel models

Multilevel models were specified in the following notation [27]:

$$ {y}_i={\alpha}_{j\left[i\right]}+{\beta}_{j\left[i\right]}{x}_i+{V}_i\gamma +{\varepsilon}_i $$

$$ \left(\begin{array}{c}\hfill {\alpha}_j\hfill \\ {}\hfill {\beta}_j\hfill \end{array}\right)\sim \mathrm{N}\ \left(\begin{array}{c}\hfill {\mu}_{\alpha}\hfill \\ {}\hfill {\mu}_{\beta \prime}\hfill \end{array}\ {\displaystyle \sum }\ \right) $$

In the equation, *y*_*i*_ represents self-rated health as outcome. j [*i*] - maps individuals to country-years. *α*_*j*[*i*]_ is the intercept, *β*_*j*[*i*]_ the random slope (logit rank of income). *γ* represents all fixed effects of subsequently added control and explanatory variables to the model: gender, age, logit rank of social origins factor, logit rank of income, education, occupation (Model 1), plus country-level of living and income inequality (Model 2), plus interactions logit rank of income with Gini (Model 3a), and logit rank of social origins with Gini (Model 3b), and *ε*_*i*_ as error term. *α*_*j*_, *β*_*j*_ are normally distributed with mean *μ* and covariance matrix *Σ*. While the terminology of multilevel models requests ‘fixed effects’ in contrast to random effects, the cross-sectional design of our study obviously does not allow causal inferences.

Multilevel models used the logit rank transformed outcome of self-rated health. Models with the ordinal outcome of health, and with dichotomized logit mixed models led to similar results. Models were run with country-year as second level since Gini and level of living diverged between measurements (2005 and 2011). Analyses with country as second level and survey year as control variable led to similar results. As overall associations of health gradients and contextual information per survey year were similar, Figures were collapsed across survey years, and data points specify both country and year of survey (’05 and ’11). A random intercept was included in all analyses. Random sloped of income were entered in all models to assess if the association of income and health varied significantly across countries. Specifying different random slopes of income, social origins, and logit rank of education led to similar model fits. All individual- and country-level variables were centered and subsequently entered as fixed effects to estimate their associations with self-rated health. Subsequently, fixed effects of individual-level variables age, gender, and stratification variables were entered (Model 1). In a next step, country-level explanatory variables level of living and level of inequality were entered (Model 2). Lastly, cross-level interaction of income inequality (Gini) with logit rank of income (Model 3a) and with logit rank of origins were entered (Model 3b, see Table 1). Additional interactions with level of living were not significant and were left out of the equation. Three-way interactions of age, income and Gini were not significant (results available upon request). Model fits were compared with deviances of the models and the Bayesian Information Criterion (BIC).

**Table 1:** Coefficients for All Individual-Level Variables (Model 1), + Country-Level Variables (Model 2), and Income*Gini (Model 3a) and Origins*Gini Interaction (Model 3b) for *N* = 299,770 Individuals

Logitrank of Health	Model 1	Model 2	Model 3a	Model 3b
Female	−0.110***	−0.109***	−0.109***	−0.109***
Age in decades	−0.424***	−0.424***	−0.424***	−0.424***
Origins	0.019***	0.019***	0.019***	0.019***
Education (ref. 1)	0	0	0	0
Edu 2	0.190***	0.190***	0.190***	0.188***
Edu 3	0.301***	0.301***	0.301***	0.301***
EGP Class (ref. 1)				
2	−0.064***	−0.064***	−0.064***	−0.064***
3	−0.111***	−0.111***	−0.111***	−0.112***
4	−0.165***	−0.165***	−0.165***	−0.163***
5	−0.257***	−0.257***	−0.257***	−0.258***
6	−0.325***	−0.325***	−0.325***	−0.325***
Income (logitr)	0.101***	0.100***	0.0947***	0.0942***
Gini (centered)		−3.282**	−2.898*	−2.900*
Country level of living		0.373***	0.372***	0.372***
Interac. Income/gini			−0.415**	−0.456***
Interac. Origins/gini				0.253***
Constant	0.057	−0.058	−0.051	−0.051
Random intercept lns1_1_1	−3.420***	−3.420***	−3.558***	−3.562***
Random slope lns1_1_2	−0.948***	−1.408***	−1.410***	−1.411***
Residual	0.374***	0.374***	0.374***	0.374***
N	299,770	299,770	299,770	299,770

Note. *p< 0.05. ***p < 0.001. Education: 1 – up to lower secondary, 2 – up to higher secondary, 3 – tertiary. EGP class: 1 – Higher level professionals, managers and entrepreneurs; 2 – lower level professionals; 3 – routine non-manual workers; 4 – small self-employed; 5 – skilled manual workers & super; 6 – semi- and unskilled manual workers & agricultural labourers. We display constant, random intercept, random slope, level 1 residuals

Results from the mixed model were graphed by post-estimating BLUPs (Best Linear Unbiased Predictors) of random slope and standard error with the Stata ‘reffects’ and ‘reses’ command. BLUP of intercept translates as median level of health, and random slope of income as steepness of the income-health gradient per country. The steeper the slope, the stronger the health gap between the poorest and the richest percentiles of the distribution. All analyses were carried out with Stata version 13.

## Results

### Descriptive statistics

The initial dataset consisted of 422,400 cases in 2005 and 432,827 cases in 2011. Many cases however could not be used for this study: Missing information for both father’s and mother’s education were large across all countries and survey years and ranged from 56 % in the Swedish 2011 sample to 91 % in the Czech 2005 and Danish 2005 sample. After excluding participants with missing information on the origins variables, a sample size of 300,787 remained. After excluding participants with missing cases on sociodemographic, occupation and education variables, a sample of a total of 299,770 participants in 18 countries was retained.

**Fig. 1:**
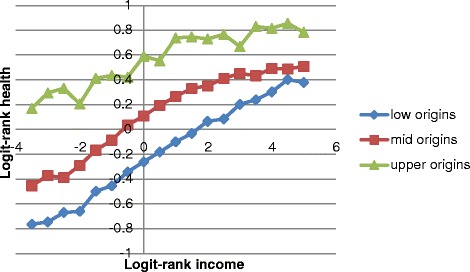
Associations of logit-rank income and logit-rank health, separate for low social origins (lower quartile of origins factor distribution), mid social origins (26th percentile to 96th percentile of origins factor), and upper social origins (upper 3 % of the origins factor)

The stratification variables were moderately but not overly correlated so that simultaneous analysis was possible (Pearson’s correlation of logit rank transformed social origins and income *r*_*P*_ = 0.20, *p* < 0.001; Spearman’s correlation of education and occupation *r*_*S*_ = −0.54, *p* > 0.001; Spearman’s correlations of education and occupation, respectively, with logit rank transformed social origins and income −0.37 < *r*_*S*_ < 0.36, *p* < 0.001). After logit-rank transforming health, income, and social origins, the advantage of using logit-rank over traditional measures is illustrated in Fig. 1: Across countries and survey years, health increases linearly with income for different levels of social origins. Higher social origins reflect better health-per-income rank on all positions of the income distribution. Health linearly increased per income quintiles per country and survey year (Appendix Fig. 8) and per social origins quintiles per country and survey year (Appendix Fig. 9). The overall association between health and level of income inequality was negative, suggesting that health is worse in more unequal countries (Fig. 3).

### Multilevel models

All stratification measures contributed independently to health, but with health slopes being differently steep. For all stratification measures the health gradient was linearly increasing. Being female was associated with worse health, being older contributed to average health decline of −0.42 per decade. Education and occupation contributed most to health, but also income and social origins after entering all other variables. Gini was negatively related with health, whereas level of living was positively related with health (Table 1). Cross-level interactions of logit rank of income with Gini were unexpectedly negatively significant (*r* = −0.42), logit rank of origins with Gini positively significant (*r* = 0.25).

**Fig. 2:**
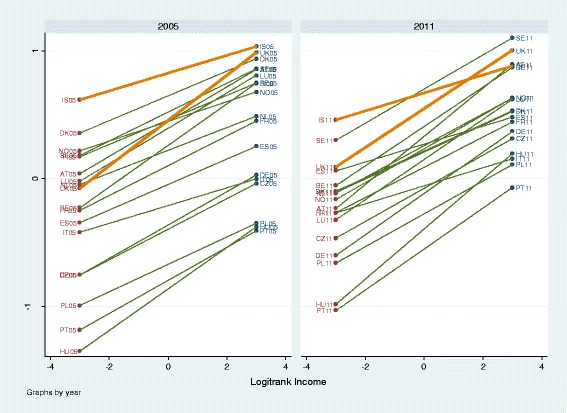
Predicted residual intercepts and slopes (BLUPs) of the “empty” model (no controls) per country and survey year

Figure 2 displays the gross income-health gradient as predicted by the model without controls. The profiles of United Kingdom and Iceland are printed in orange, showing that despite steep health gradients such as in the United Kingdom, health of the richest are below those of Iceland, a more egalitarian, whereas health of the poorest in the United Kingdom is much worse than of those of Iceland.

**Fig. 3:**
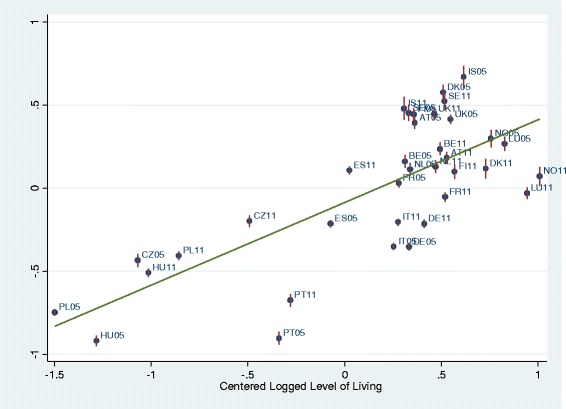
Predicted residual health slopes (BLUPs) of model 2 (all individual-level variables, no country-level controls) per country and survey year against national level of economic development

**Fig. 4:**
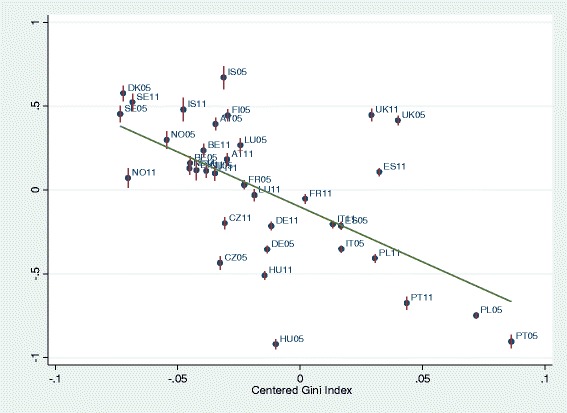
Predicted residual health slopes (BLUPs) of model 2 (all individual-level variables, no country-level controls) per country and survey year against national level of income inequality (Gini)

**Fig. 5:**
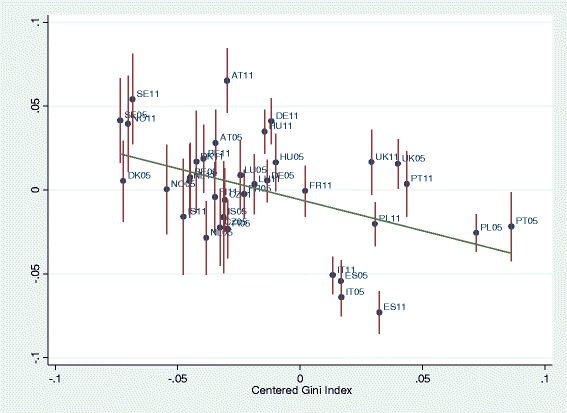
Association of income-health gradient and level of inequality

**Fig. 6:**
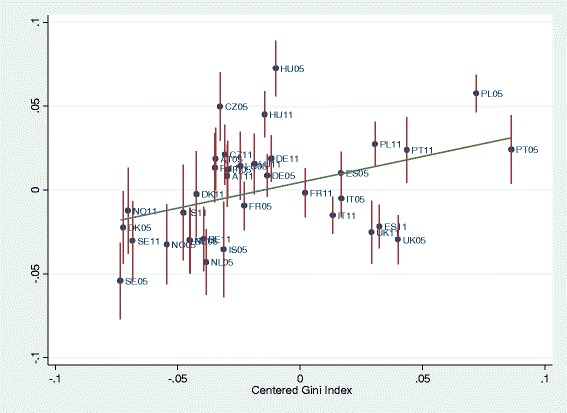
Association of origin-health gradient and level of inequality

BLUPs were used to graph income-health gradients per country depending on level of living and of income inequality per country, and to graph the level of health per country depending on level of living and of income inequality. First, the predicted residuals of intercepts and slopes (BLUPs) by country-years of model 2 (all individual-level variables) were plotted against national level of living and Gini indices. National level of living was associated with better health (Fig. 3). As expected, higher inequality in the country was associated with lower health (Fig. 4). Figure 5 graphs the unexpected negative association of income-health gradient and Gini, showing that in higher income inequality countries, the income was less strongly related with health. For Fig. 6, we ran a model with random slope of origins and post-estimated BLUPs of the origin-health gradient. This analysis shows the expected finding that, in higher income inequality countries, origins are stronger associated with health.

### Additional model validation

Models with random slope of social origins and with two slopes of income and social origins were run but did not lead to model fit improvements or additional analytical insight. Models with the ordinal or a dichotomized measure of self-rated health as outcome led to practically similar results. Analyses using the logit ranked variable of health were further validated by bootstrapping results (100 repetitions). Entering the original variables of the social origins composite separately as fixed effects (mothers’ and fathers’ education and occupation) and logit rank of education as explanatory variables led to similar results.

In additional analyses, limiting long-standing illness (LLSI; dichotomous indicator with 1 being absence of LLSI) was used as outcome, one of the few additional health variables available in EU-SILC. Generally, associations of socio-demographic variables and income with LLSI were similar in size and direction of the association, but the origins factor, Gini and Level of Living were only marginally associated with LLSI. Cross-level interactions of income and origins with Gini were again practically similar to those obtained with the logit-rank and ordinal measures of health (results available on request).

## Discussion

### Explanation of findings

Our paper provides evidence on how different measures of stratification can explain differences in health in a large sample of 18 EU countries. We replicate the positive association of income and health [28] and the negative association of income inequality and health [29–32]. By applying the innovative logit rank tool to income, analyses confirmed a linearly increasing health gradient even at the very extremes of the distribution, suggesting that it is always better in terms of health to have a higher socioeconomic position, even for individuals at the very bottom or very top of the distribution. Similar to Wilkinson and Pickett (2010) who basically argue that inequality is systemic stress for the population and leads to decreases in population health, our findings suggest that in Europe higher income is good for health, but inequality as such threatens country’s average health level. The finding that income inequality is negatively related to average health suggests that nations with higher inequality levels could be neglecting public health issues which leads to lower health for the general population.

In line with our findings, social origins have been shown to have important influences on adult health [28, 33], and our study extends these findings insofar as even after detailed controls for current socioeconomic position, social origins are still associated with self-rated health. Further, income inequality increased the association of social origins with health across countries. These findings show that even in meritocratic countries, there is intergenerational transmission of health via parental socioeconomic status after controlling current measures of individual socioeconomic position (which may also be influenced by parental socioeconomic status). A more unexpected pattern was that with higher income inequality, income-health gradients were attenuated. This paradoxical finding can be explained by comparatively high Northern European income-health gradients and, conversely, low income-health gradients of Italy, Spain and Poland. Indeed, our findings echo the European income-health estimates of Beckfield and Olafsdottir who investigated low-income disadvantages and high-income advantages in health in the World Values Survey: While Beckfield and Olafsdottir report for Norway both high low-income disadvantage AND high-income advantage for health, our analyses produce comparatively large health gradients for Norway, meaning that within the low range of income distribution, health levels vary greatly [34]. The negative association of income inequality with the income-health association could also be due to sampling specificities in this sample of 18 high-income countries – as was shown, only few countries show associations in the expected direction of positive associations of income inequality and health gradient such as United Kingdom and Iceland. This ‘public health puzzle’ or ‘paradox’ of high inequalities in egalitarian Northern European regimes has been noted several times in the literature and common explanations of life course, health selection and other causes can only partly explain this finding [35, 36]. However, it is more likely that unobserved country characteristics have produced this finding: Other country characteristics with evidence of producing differences in the health gradient are welfare regimes [37–41], political systems [42], health expenditures and labour market conditions [43], public versus private-based healthcare systems [44], social expenditures [45], and health policy performance [46]. The pattern of our findings does not obviously point to one of those explanations. It may be possible though that in this sample of respondents from Northern European countries, low levels of income inequality mask large health gaps between lower and higher socioeconomic groups, and may be a result of class-specific attitudes and (health) behaviors [3, 47]. Concerning the sample of Southern European countries Portugal, Poland, Italy, and Spain, all with a high rate of members of the Catholic church compared to the other countries in this study, it could be argued – as all countries with this positioning of health gradient and income inequality are known for their familialistic welfare system – that high income inequality is buffered by traditional family systems and may increase especially health of lower socioeconomic groups. This familialistic protection, perhaps motivated by religious practices, may be one of the reasons why the health gradient in those countries was not as steep as expected. However readers should note that this study was not designed to assess the influence of country-level familialism on the health gradient, and further studies should move forward to possible explanations for the low association between income inequality and health gradient. Another possible explanation would be reporting differences in health, which has been shown e.g. with health vignettes from SHARE [48], but which do not reflect the pattern of educational inequalities we find in the EU-SILC data.

Additional analyses with limiting long-standing illness (LLSI) as outcome confirmed overall robustness of our results, although the general picture with this indicator was less clear compared with the analyses on self-rated health. This is not surprising considering that cross-national patterns with LLSI have been called enigmatic [49]. Further, the association between self-rated health and disability gradient has been shown to be subject to welfare state differences itself [41]. Further studies should explore in more detail the associations of income inequality, income-health gradient and limiting long-standing illness, preferably with a more age-heterogeneous sample.

### Strengths and limitations

Methodologically, strength of the study is the use of logit rank transformation to detect detailed health gradients of social origins and income, providing a tool to compare health and other outcomes even at the very extremes of the distribution. Another strength is the exploitation of the estimates of residual slopes and intercepts to assess the health gradients per country as a tool to detect relative position of countries in terms of health gradient and to assess its associations with other variables of interest. Conceptually, our paper makes use of a large dataset to assess the role of social origins on health, and finds that even after controlling for other indicators of socioeconomic class and position, origins still play a role for health.

One limitation of this contribution is the lack of possibilities to draw causal inferences due to the cross-sectional nature of this study. Caution is warranted in interpreting the specificities of the origin-health gradient, both due to massive attrition with regard to missing information in the EU-SILC intergenerational module and the question of representativeness of social origin information in the remaining dataset [21]. Detailed analyses of the Intergenerational Module point to non-random missing data in respondents with low-educated parents, especially of the Scandinavian countries and the UK [21]. It is likely that missing information of those respondents with low-educated parents may have downwardly biased our results and health gradients in a hypothetical dataset without missing data would be steeper. Further, health gradients may also be differently associated with income inequality if missing information across countries was non-random. Further comparative studies with datasets with preferably fewer missing data on social origins are warranted to replicate our findings. However, the EU-SILC data still provide a unique detailed assessment of social origins in a comparative perspective. We tentatively conclude for this paper that the impact of social origins is significantly higher in high income inequality countries.

### Implications for policymaking

Implications for policymaking derived from this study mainly regard establishing and maintaining support systems for the most vulnerable individuals with low income. Low overall income inequality may disguise health problems of groups with low socioeconomic status. Therefore, efforts to improve population health extend income redistribution and have to be addressed specifically: health problems and related problems of health behaviors, lifestyle etc. need to be closely monitored and improved, especially of the at-risk individuals with lowest income. Efforts to raise educational attainment and professional training of the general population should maximize potential of individuals despite intergenerational transmission of status and, in turn, may positively influence population health.

## Conclusions

In this sample of European countries, we find intergenerational transmission of health via socioeconomic position and social class of parents even after controlling for powerful indicators of current socioeconomic class and position. Income inequality is generally bad for health, but with higher income inequality, the health gradient shows differing associations across countries, with steep health gradients despite low income inequality in Northern European countries, and low health gradients despite high income inequality in Southern European countries and Poland. We conclude that further unobserved country characteristics such as familialistic welfare systems shape associations of income inequality and health gradient, and in order to explain health of lower socioeconomic groups, additional variables have to be observed. Health-related policymaking should go beyond ensuring low income inequality and specifically address health problems of groups with low socioeconomic status.

## Figures and Tables

**Fig. 7:**
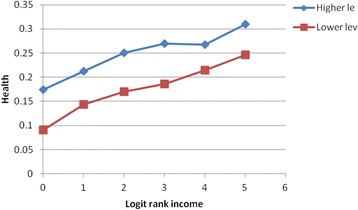
Association of logit rank of income with health for higher level service class (EGP = 1) and lower level service class (EGP = 2)

**Fig. 8:**
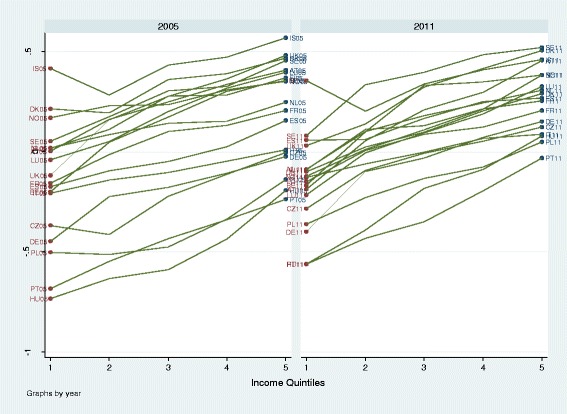
Distribution of health per income quintiles per country and survey year

**Fig. 9:**
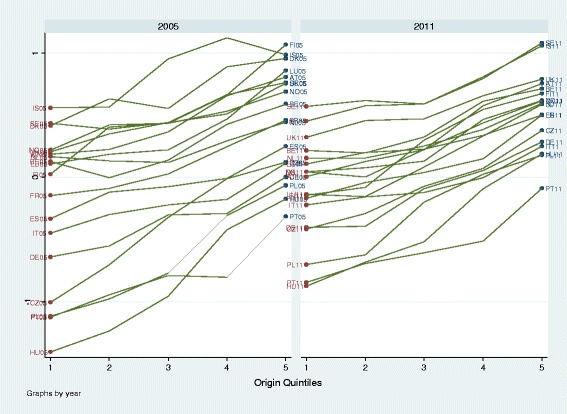
Distributions of health per origins quintiles per country and survey year
